# Patient satisfaction & use of health care: a cross-sectional study of asylum seekers in the Freiburg initial reception centre

**DOI:** 10.1186/s12913-020-05579-7

**Published:** 2020-08-03

**Authors:** Annabelle J. Bockey, Aleš Janda, Cornelia Braun, Anne-Maria Müller, Katarina Stete, Winfried V. Kern, Siegbert R. Rieg, Berit Lange

**Affiliations:** 1 Department of Medicine II, Division of Infectious Diseases, University Hospital Freiburg, Freiburg, Germany; 2 PhD Programme “Epidemiology” Braunschweig-Hannover, Helmholtz Centre for Infection Research, Inhoffenstraße 7, 38124 Braunschweig, Germany; 3 Department of Epidemiology, Helmholtz Centre for Infection Research, Inhoffenstraße 7, 38124 Braunschweig, Germany; 4 Centre for Paediatrics and Adolescent Medicine, University Medical Centre, Medical Faculty, University of Freiburg, Freiburg, Germany; 5 EA Clinic for Refugee Medicine, Freiburg, Germany; 6 Department of Pediatrics and Adolescent Medicine, University Medical Center Ulm, Ulm, Germany; 7 Centre for Mental Health, Department of Psychosomatic Medicine and Psychotherapy, Medical Centre – University of Freiburg, Faculty of Medicine, Freiburg, Germany; 8 Helmholtz Centre for Infection Research, Braunschweig, Germany

**Keywords:** Delivery of health care, Patient satisfaction, Refugees & Asylum Seekers, Germany

## Abstract

**Background:**

In response to a high number of incoming asylum seekers and refugees (AS&R) in Germany, initial reception centres were established to provide immediate shelter, food and health support. This study evaluates the satisfaction with and use of the health care available at the Freiburg initial reception centre (FIRC) where an integrated health care facility (ICF) was set up in 2015.

**Methods:**

We assessed use and satisfaction with health services available to resident AS&R within and outside the FIRC in a cross-sectional design. Data were collected in 2017 using a questionnaire with both open and closed ended items.

**Results:**

The majority of 102 included participants were young (mean age 24.2; 95%CI 22.9–25.5, range 18–43) males (93%), from Sub-Saharan Africa (92%). High use frequencies were reported from returning patients of the ICF; with 56% fortnightly use and 19% daily use reported. The summary of satisfaction scores indicated that 84% (CI95 76–89%) of respondents were satisfied with the ICF. Multivariate analysis showed female gender and non-English speaking as risk factors for low satisfaction. Outside the FIRC, the satisfaction scores indicated that 60% of participants (95%CI 50–69%) were satisfied with the health care received.

**Conclusion:**

Our study shows that AS&R residing in the FIRC are generally satisfied with the services at the ICF, though strategies to enhance care for females and non-English speakers should be implemented. Satisfaction with health care outside of the FIRC was not as high, indicating the need to improve quality of care and linkage to regular health care services.

## Background

In 2015, 2016 and 2017 more than 1.4 million persons applied for asylum in Germany [1]. In Germany accommodation of asylum seekers and refugees (AS&R) is a federal responsibility, while the asylum procedure itself is managed by a national institution, the Bundesamt für Migration und Flüchtlinge (BAMF). Most federal states in Germany provide first accommodation with and after registration in initial reception centres [2]. In dealing with this increased arrival of AS&R in 2015 and 2016 most states in Germany established or updated initial reception centres to provide food, shelter, health and other support in the initial months after arrival [2]. For many AS&R, accommodation in initial reception centres is compulsory for the duration of the application process [2].

Following the initial health screening, depending on the legal residence status of the AS&R and a waiting time regulation, access to health care for AS&R in Germany is restricted under the Asylum-Seekers’ Benefits Act (AsylbLG) for the first 15 months of their residence in Germany [3]. For those awaiting decisions on their asylum application, those denied asylum but not yet repatriated and those granted a temporary residence permit on humanitarian grounds, health care is restricted to emergency medical care, treatment for acute conditions and necessary preventative measures [3, 4]. AS&R are a highly vulnerable group, and the need for appropriate and accessible health services are essential in providing mental and physical support tailored to the situation of each AS&R. Restrictions to health care not only negatively affects the health of AS&R, but has been shown to have a higher financial burden to the German health system [5].

The provision of health care services varies amongst reception centres throughout Germany, but most reception centres provide basic services within the centres and also refer patients for emergencies or specialist appointments to regular health services in Germany [6] [4]. Initial reception centres without health facilities provide the resident AS&R with health insurance vouchers when necessary, whereby treatment is obtained at regular health facilities [7]. While some federal states provide regular health insurance cards to AS&R, this is currently only five of the sixteen German states, including Berlin, Brandenburg, Bremen, Hamburg and Schleswig-Holstein [5].

In the context of the rapidly growing number of newly arrived AS&R, the regional state authority responsible (Regierungspräsidium) in Baden Württemberg established a new reception centre in Freiburg, with a residential capacity for 500 AS&R. Healthcare on site is offered by a university hospital led clinic that is coordinated by the Department of Infectious Diseases at the University Hospital Freiburg. It is an integrated care model encompassing adult, paediatric and mental health services on site. Where necessary, other specialised medical experts visit the health clinic, or patients are referred to other cooperating departments of the University Hospital for specialist care. Provisionally since November 2015 and in full service since March 2016, the clinic provides consultations on weekdays to approximately 20 to 30 patients daily, whereby at least one doctor and one nurse are on site, while a psychologist offers twice weekly appointments [8].

Limited research has been undertaken to evaluate the health services available to AS&R in reception centres in Germany, especially from the patient perspective. In Germany, the focus has previously been on the availability of AS&R services for health care provision [4, 5]. Some studies have been conducted in initial reception centres, yet only public health authorities and not AS&R were surveyed. The results of these studies reported variable care for AS&R in Germany, where the use of standards and general guidelines were infrequent [9, 4].

Patient satisfaction is an important component of evaluating health services, and despite being a key component to Germany’s general monitoring of health services, is yet to be included in evaluating the health care services provided to AS&R [10, 11]. Researching patient satisfaction can be a valuable tool in predicting health related behaviour, influencing the use of health care, evaluating difficulties to obtaining care and assessing communication patterns [10]. Surprisingly, few studies have taken patient satisfaction into account when assessing the health care available to vulnerable groups, despite the additional health needs of vulnerable populations and the health of migrant populations listed as a high priority in Germany [12]. A PubMed scan of studies on patient satisfaction of migrants found few comparable papers. One related quantitative study of AS&R in an initial reception centre in Wales assessed satisfaction, health literacy and health problems, but in relation to the initial health assessment [13]. The study reported positive satisfaction, yet low health literacy [13]. An Australian study assessed patient satisfaction of an integrated healthcare service for AS&R, where high levels of satisfaction were reported, especially in relation to the availability of bicultural staff, translation services and the integration of care [14]. Two studies on refugees in the United States [15, 16] reported overall satisfaction for health care, with one reporting lower satisfaction for phone translation and time with the doctor [15], while the second reported fluency of English and level of education inversely associated with satisfaction [16]. Despite evidence showing many barriers to health care access, low health literacy and differing levels of satisfaction, there is a significant gap in research relating to patient satisfaction amongst AS&R. This study aims to address this gap, by evaluating the health care services available to asylum seekers staying in an initial reception centre in Freiburg through a cross-sectional pilot study.

The main objective of the study was to describe the use of health care facilities by FIRC resident AS&R both inside and outside the FIRC. Other objectives were to assess the satisfaction of resident AS&R staying at the FIRC with the ICF, as well as their satisfaction with health care outside of the FIRC and identify determinants for low satisfaction.

## Methods

### Study design

A cross-sectional pilot study under STROBE (STrengthening the Reporting of OBservational studies in Epidemiology) guidelines [17] was used for this research to assess the satisfaction with health services available to asylum seekers staying at the Freiburg initial reception centre (FIRC).

### Setting

Located in the city of Freiburg in the state of Baden-Württemberg, Germany, the research was undertaken at the local FIRC, which has an integrated health facility (ICF) available on weekdays, as previously described [8].

### Participants

Participants who took part in the study were AS&R staying at the FIRC who were older than 18 years of age. Participants were recruited by Annabelle Bockey at the FIRC. Fitting to the varying levels of literacy at the FIRC, the questionnaires were conducted as face-to-face interviews during the opening hours of the ICF, between 10:00 and 14:00. The questionnaire was piloted and discussed with local staff at the FIRC during May and June 2017, and recruitment was from June to August 2017, when approximately 200 AS&R were staying at the FIRC.

### Data sources

Data were collected through a questionnaire that employed intra-method mixing, a technique that uses both open and closed ended items to achieve more comprehensive data [18]. Drafted in consultation with researchers and those working at the FIRC, the questionnaire was divided into three parts to establish a) participant characteristics b) use and satisfaction with health care services within the FIRC c) use and satisfaction with health care services outside of the FIRC (for full questionnaire please see supplemental material). To establish participant satisfaction, which we define as patients’ attitudes and perception towards care, questions from the validated German ZUF-8 client satisfaction questionnaire (allowing for comparison) were used, supported by qualitative questions [11, 19, 20].

Based on the American CSQ-8 client satisfaction questionnaire, the ZUF-8 questionnaire is validated as a one-dimensional and reliable scale (Cronbach’s α = 0.90) [21]. The questionnaire is used to measure satisfaction through the use of an eight-question comprehensive self-assessment [22]. As we were primarily interested in the services provided in the FIRC all eight of the ZUF-8 questions were used to establish satisfaction of services at the ICF, while just four of the eight ZUF-8 questions were used to establish satisfaction of health care services outside of the ICF. These additional four questions allowed for comparison of satisfaction.

All questionnaires were completed anonymously, and ethical guidelines were strictly followed. A consent form was signed by each participant, which explained the study and the responsibilities of the participants and interviewer. All procedures performed in the study were in accordance with the ethical standards of the academic institution of the University of Freiburg and with the 1964 Helsinki declaration and its later amendments. Ethical clearing was provided for this study by the University of Freiburg (application number 153/17).

### Bias

In order to mitigate potential selection bias, the questionnaire was translated into five key languages spoken at the FIRC, including English, German, Arabic, French and Chinese. Due to low levels of literacy, English and German questionnaires were offered verbally as interviews, and peers were permitted to assist with the completion of questionnaires in other languages.

### Statistical methods

We performed a sample size calculation aiming at being able to detect a proportion of satisfied AS&R of 50% with a margin of error of 10% and a confidence interval of 95%, leading to a sample size calculation of 100–150 participants.

The data were collected, entered into Microsoft Excel 15.0 for processing, checked at 10 % for accuracy and analysed using Stata/IC 14 and R Studio 1.0.153. For the assessment of health care use we generated the average number of services visited per year by multiplying the monthly number of visits by 12 for each medical service used outside the FIRC, assuming similar use throughout the year. For inside the FIRC, the mean was also calculated yearly, based on the reported visits while excluding first time visits. Means, medians, standard deviation, range and confidence levels (95%) were also generated based on these annual figures. Due to the limited number of samples, the non-parametric Kruksal-Wallis test was applied to assess the difference in use frequencies.

Satisfaction data were numerated according to the ZUF-8 scoring system, whereby a number range of 1–4 was applied, with higher scores [3, 4] representing greater satisfaction. This numeration enabled a satisfaction summary range of 8 to 32 for the first section satisfaction of health care services within the FIRC, and 4 to 16 for satisfaction with health care services outside of the FIRC. To describe satisfaction, mean satisfaction scores and 95%CI were calculated, whereby mean participant scores of 2.9 or greater were considered to represent overall satisfaction (either somewhat satisfied or largely satisfied).

To assess risk factors for low satisfaction, we explored several risk factors using univariate analysis performing the Chi squared test. Building on these results, multivariate analysis through a logistic regression was performed on four risk factors together, language, gender, number of reception centres and duration of stay in the FIRC, to assess independent association on satisfaction, with a cut off of 2.9 as an indicator of satisfaction.

## Results

**Fig. 1:**
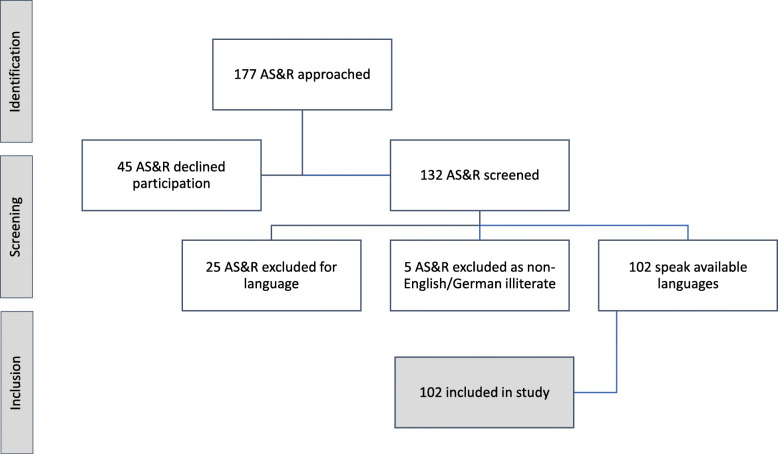
Participant Attrition

The final study population comprised of 102 AS&R staying at the FIRC. Forty-five AS&R declined participation, while eligibility criteria ruled out 30 participants (response rate 60%) (Figure 1). Of those who responded, 93% of the participants were male, and more than 70% of the participants were 25 years of age or below (mean 24.2, 95%CI 22.9–25.5, range 18–43) (See Table 1).

**Table 1:** Participant Characteristics

Characteristics	n – number	Percentage: n (%)
Gender	**99**	
Male	92	93%
Female	7	7%
Age (years)	**98**	
< 21	40	41%
21–30	39	40%
> 31	19	19%
Region of Origin	**99**	
Sub-Saharan Africa	91	92%
South and Central Asia	2	2%
Eastern Europe	3	3%
Middle East	3	3%
Language used for questionnaire	**102**	
English	94	92%
German	0	0%
French	7	7%
Arabic	1	1%
Chinese	0	0%
Duration of Stay at FIRC (months) ^a^	**93**	
< 1 month	27	29%
1–3 months	46	49%
4–6 months	10	11%
7–9 months	9	10%
> 10 months	1	1%
Number of German Reception Centres visited	**92**	
1	6	6%
2	38	40%
3	36	38%
4	12	13%

^a^at the time of the interview

Although 14 languages were reported as being spoken by the participants, the majority of questionnaires were completed in English (92%), followed by French (7%) and Arabic (1%). The participants came from 11 countries, making up five geographical regions, with most from Sub-Saharan Africa (92%). 94% of participants had previously lived in another German reception centre (mean 2.8, CI95% 2.6–3.0) and reported staying at the FIRC for a duration range of 1 day to 10 months (mean 2.6 months CI95% 1.3–3.8).

With relation to the use of health care services, of returning ICF patients, 56% of respondents reported visiting ICF at the FIRC at least fortnightly. Outside of initial reception centres in Germany, hospitals were the most reported services used, representing 47% of those who used other services. This differed to use of health services in countries other than Germany, whereby a family doctor was reported as the most frequently used in 75% of cases in their home country, and 51% of cases in other countries. A comparison of the use frequencies in different locations for any health services as well as the FIRC are displayed in Table 2, whereby use for health services in the FIRC are considerably higher than other locations. The results of the Kruksal-Wallis test strengthened these findings, as the population medians were found to be significantly different (K = 32.6, critical value = 7.8, *P* = < 0.001) .

**Table 2:** Use of Health Care Services: Number of visits/use of combined health care services per year

	Location of Health Services			
	Home Country	Country other than home or Germany	Germany (outside FIRC)	Freiburg Initial Reception Centre
**Mean**	7.7	16.6	25.3	47.4
**Median**	6.0	12.0	18.0	12.0
**Standard Deviation**	6.9	12.7	18.4	82.3
**Range**	25.0	71.0	72.0	256.0
**Minimum**	1.0	1.0	12.0	4.0
**Maximum**	26.0	72.0	84.0	260.0
**Number of responses**	59.0	45.0	28.0	96.0
**Confidence Level (95%)**	1.8	3.8	7.1	16.7

The summary of satisfaction scores for the ICF indicated that 84% of respondents were satisfied (either somewhat satisfied or largely satisfied) with the ICF (95%CI 76–89%), and only 3.1% reported being very unsatisfied. Each question in this section received a mean score of more than 3.2 (95%CI 3.02–3.85), indicating good satisfaction overall (see Table 3).

**Table 3:** Participant Satisfaction

Question	n	Very satisfied (4)	Mostly satisfied (3)	Somewhat unsatisfied (2)	Unsatisfied (1)	Mean	Standard Deviation	Margin of Error	Upper Limit 95% CI	Lower Limit 95%CI
Q 1. How would you judge the quality of the treatment you received?	99	35%	52%	12%	1%	3,21	0,69	0,07	3,35	3,08
Q 2. Did you receive the kind of treatment you wanted?	99	49%	28%	15%	7%	3,20	0,95	0,10	3,39	3,02
Q 3. To what extent did our clinic meet your needs?	99	52%	27%	18%	3%	3,27	0,87	0,09	3,44	3,10
Q 4. Would you recommend our clinic to a friend or relative if he/she would need similar help?	97	76%	15%	4%	4%	3,64	0,75	0,08	3,79	3,49
Q 5. How satisfied are you with the extent of the help you received?	97	56%	30%	7%	7%	3,34	0,90	0,09	3,52	3,16
Q 6. Were you satisfied making the initial appointment at the Initial reception centre?	97	84%	10%	4%	2%	3,75	0,63	0,06	3,88	3,63
Q 7. Did the treatment you received help you deal with your problems more appropriately?	91	66%	22%	11%	1%	3,53	0,74	0,08	3,68	3,38
Q 8. How satisfied are you with the treatment that you have received, by and large?	94	53%	21%	24%	1%	3,27	0,87	0,09	3,45	3,09
Q 9. Would you return to our clinic if you needed help?	97	82%	8%	8%	1%	3,72	0,66	0,07	3,85	3,59
Q 16. (Medical Care Outside) How would you judge the quality of the treatment you received?	86	22%	48%	26%	5%	2,87	0,81	0,09	3,04	2,70
Q 17. (Medical Care Outside) Did you receive the kind of treatment you wanted?	86	31%	30%	20%	19%	2,74	1,10	0,12	2,98	2,51
Q 18. (Medical Care Outside) To what extent did the other clinic meet your needs?	85	24%	25%	32%	20%	2,52	1,06	0,12	2,74	2,29
Q 19. (Medical Care Outside) How satisfied are you with the extent of the help you received?	83	36%	25%	18%	20%	2,77	1,15	0,13	3,02	2,52

The summary of the four satisfaction questions related to other health care services in Germany indicated that 60% of participants were satisfied with the health care services in Germany outside of the FIRC (95%CI 50–70%). The means for each question remained between the satisfaction scores of 2.5 and 3 (95%CI 2.29–3.04). The comparison of the patient satisfaction scores in the ICF and health care outside the FIRC reveals that there is a trend of higher satisfaction reported in the ICF. Furthermore, as the confidence intervals do not overlap, this indicates that there is evidence that AS&R living in the FIRC viewed outside care as less satisfactory (see Table 3).

The univariate analyses found English speakers to have higher odds of having higher satisfaction scores (OR 13 *P* = < 0.0005). Other variables that were tested, such as gender, number of reception centres in Germany and duration of stay at the FIRC did not reach statistical significance (*P*= > 0.05).

The multivariate analysis showed males to have higher odds ratio for higher satisfaction (*P* = < 0.005, OR 31.28, CI95% 2.59–377.20), and there was higher satisfaction amongst English speaking participants compared to non-English speaking participants (*P* = 0.012, OR 29.84, CI95% 2.12–419.97).

The supporting open-ended questions frequently mentioned broadly “treatment issues” as a cause for low satisfaction in receiving the kind of treatment wanted at the ICF. Government restrictions were also mentioned, along with treatments not within the scope of the services able to be provided, for example dental, operations and physiotherapy. The causes of difficulties experienced in accessing care were similar, in addition to: waiting time, referrals, opening hours and multiple transfers between reception centres causing missed specialist appointments. Suggestions to overcome these barriers were removing government restrictions, more staff and an appointment schedule. Other important problems that were reported included issues with treatment, especially dental, eye or ear care, and dissatisfaction with the living conditions and food provision.

## Discussion

High non-participation was experienced in recruiting for the study, and the recruited participants were largely homogenous with respect to their gender, age and origin. Participating AS&R reported a high frequency of engagement with the ICF, while a less frequent use of other health care facilities, as these were only used in case of referrals or when the ICF was closed. Close proximity to the ICF at the FIRC is likely to have contributed to this high use, as well as the fact that recruitment for participation in the study was undertaken mostly within the ICF.

Due to the extreme vulnerability of AS&R, from often traumatic experiences in their home country or on their journey to Germany, or both, combined with the fragile situation of applying for asylum or appealing a negative response for the asylum application, it was not surprising that many did not want to participate in the study. Although the participant cohort was representative of the AS&R staying in the FIRC with regard to gender and country distribution at the time, the sample does not necessarily reflect the characteristics of all AS&R across Germany, as in 2017 more than 44% of AS&R came from Syria, Iraq and Afghanistan, 60.5% were male and 30.3% between the ages of 18–30 [1] [23]. This is largely due to the streaming of AS&R with a low success rate, and different German states specialising in different countries or regions to aid the application process [24]. This can be seen in the large number of participants from sub-Saharan Africa and the largely male cohort. Due to the recruitment at the ICF, selection bias must also be acknowledged, and although strategies to mitigate selection bias were applied, social desirability bias is difficult to negate and may have been exacerbated by the use of face-to-face interviews to ensure illiterate AS&R were not excluded from the study. Nonetheless, as the interviewer was not a part of the ICF or the FIRC and conducted the interviews in private, we hope the social desirability bias was somewhat neutralised.

The main satisfaction results illustrate that the participants experienced general satisfaction with the ICF. Lower scores were recorded for items that mentioned “treatment”, with treatment also listed as key concern in the open questions. Results for medical care outside the FIRC indicated that participants were slightly less satisfied with care; though in part this may be due to social desirability bias, it is also likely a result of more specialised and migrant-sensitive care offered in the FIRC. This result is similar to a United States (US) study on Vietnamese Refugees, whereby greater satisfaction was found with the specialised treatment unit for refugees, rather than regular services [16]. However, it must be noted that in Freiburg, outside care facilities are accessible to the patients by referral through the treating doctor in the ICF (except emergencies out of hours). As the ICF is intended for all health care needs, referrals are reserved for more severe or specialised cases. Therefore, it is understandable that some of the dissatisfaction reported for outside care is attributable to these factors, as specialised care often takes longer, is more complicated, and is partly restricted by law. Nevertheless, the results are comparable to other patient satisfaction studies in Germany, which indicate more than 59% of respondent’s as satisfied with primary care [25, 26].

Outcomes indicate non-English speaking and female gender as possible determinants for lower satisfaction, highlighting the need for gender appropriate care and better communication. Despite being dissimilar to a US study on refugees where fluency of English was inversely associated with satisfaction [16], the relationship between language and satisfaction was not unexpected. Due to the language restrictions of the staff at the FIRC and without a permanent interpreter, language remains a barrier to non-English speaking patients. Though costly, a possible solution to this would be the application of video interpreting service. Although we acknowledge that the non-English speaking and female participants were small, we believe the results are indicative of the perspective of these groups who remain in the minority at the FIRC, and argue that additional services should be made available to assist non-English speaking as well as female AS&R.

The open question results additionally raise concerns related to the situation at the FIRC largely out of the control of the ICF, including health care restrictions and the other services within the camp (including food). Responses also show that the expectations of AS&R were often unrealistic for a primary health care service in Germany, whereby certain treatments or medications are rarely available in this setting. Furthermore, as the provision of health care is restricted to AS&R under the Asylum Seekers’ Benefits Act, some level of dissatisfaction with treatment was expected. The responses in the open-ended question illustrate that the restrictions have an impact on the satisfaction with health care and can be a barrier to health access. Research on these health care restrictions support this argument, where it is shown that the restrictions act as a barrier to health care access and are more costly than regular health access [5, 9].

## Conclusions

Notwithstanding the limitations, this study is relevant as an indicator of the quality of health services available for AS&R staying at the FIRC. The study more generally also reflects an important view of the patient satisfaction, which has been underreported in studies of AS&R health care in Germany [10, 21]. Overall, there is a strong need to improve quality of care particularly to females and non-English speaking AS&R. Nonetheless, the high use and solid satisfaction of the ICF indicate that the services delivered at the FIRC are positively received. The difference in satisfaction scores inside and outside the FIRC supports the need for improved communication, linkage and integration of care to regular health care services for AS&R in Germany. The study is easily reproduced and could be used to gain insight into different health care services available to AS&R.

To strengthen these findings, further research is required that looks at linking specific care situations, including health problems, age and other variables. Multi-centre research would also allow for clear comparisons between different approaches to the provision of health care to AS&R. Such research would not only be useful in identifying the health needs of this marginalised group, but also methods to improve health care access and use in centralised care worldwide.

## Supplementary information

**Additional file 1.** Health Care Questionnaire.

## Data Availability

All data generated or analysed during this study are included in this published article [and its supplementary information files].

## Abbreviations

AS&R: Asylum Seekers and Refugees
FIRC: Freiburg Initial Reception Centre
ICF: Integrated Care Facility
