# Supplementary material for: Patient satisfaction & use of health care: a cross-sectional study of asylum seekers in the Freiburg initial reception centre
Source: BMC Health Serv Res. 2020 Aug 3;20:709. doi: 10.1186/s12913-020-05579-7 (PMC7397598; doi:10.1186/s12913-020-05579-7)
Supplement: Supplementary file 1 — Additional file 1. Health Care Questionnaire. [file 12913_2020_5579_MOESM1_ESM.docx]

# Supplemental: Health Care Questionnaire
